# Association of Display of Patient Photographs in the Electronic Health Record With Wrong-Patient Order Entry Errors

**DOI:** 10.1001/jamanetworkopen.2020.19652

**Published:** 2020-11-11

**Authors:** Hojjat Salmasian, Bonnie B. Blanchfield, Kelley Joyce, Kaila Centeio, Gordon B. Schiff, Adam Wright, Christopher W. Baugh, Jeremiah D. Schuur, David W. Bates, Jason S. Adelman, Adam B. Landman

**Affiliations:** 1 Department of Quality and Safety, Brigham and Women’s Hospital, Boston, Massachusetts; 2 Division of General Internal Medicine, Brigham and Women’s Hospital, Boston, Massachusetts; 3 Harvard T.H. Chan School of Public Health, Boston, Massachusetts; 4 Vanderbilt University Medical Center, Nashville, Tennessee; 5 Department of Emergency Medicine, Brigham and Women’s Hospital, Boston, Massachusetts; 6 Department of Emergency Medicine, Rhode Island Hospital, Providence; 7 Alpert Medical School of Brown University, Providence, Rhode Island; 8 Department of Quality and Safety, NewYork-Presbyterian Hospital, New York, New York

## Abstract

**Importance:**

Wrong-patient order entry (WPOE) errors have a high potential for harm; these errors are particularly frequent wherever workflows are complex and multitasking and interruptions are common, such as in the emergency department (ED). Previous research shows that interruptive solutions, such as electronic patient verification forms or alerts, can reduce these types of errors but may be time-consuming and cause alert fatigue.

**Objective:**

To evaluate whether the use of noninterruptive display of patient photographs in the banner of the electronic health record (EHR) is associated with a decreased rate of WPOE errors.

**Design, Setting, and Participants:**

In this cohort study, data collected as part of care for patients visiting the ED of a large tertiary academic urban hospital in Boston, Massachusetts, between July 1, 2017, and June 31, 2019, were analyzed.

**Exposures:**

In a quality improvement initiative, the ED staff encouraged patients to have their photographs taken by informing them of the intended safety impact.

**Main Outcomes and Measures:**

The rate of WPOE errors (measured using the retract-and-reorder method) for orders placed when the patient’s photograph was displayed in the banner of the EHR vs the rate for patients without a photograph displayed. The primary analysis focused on orders placed in the ED; a secondary analysis included orders placed in any care setting.

**Results:**

A total of 2 558 746 orders were placed for 71 851 unique patients (mean [SD] age, 49.2 [19.1] years; 42 677 (59.4%) female; 55 109 (76.7%) non-Hispanic). The risk of WPOE errors was significantly lower when the patient’s photograph was displayed in the EHR (odds ratio, 0.72; 95% CI, 0.57-0.89). After this risk was adjusted for potential confounders using multivariable logistic regression, the effect size remained essentially the same (odds ratio, 0.57; 95% CI, 0.52-0.61). Risk of error was significantly lower in patients with higher acuity levels and among patients whose race was documented as White.

**Conclusions and Relevance:**

This cohort study suggests that displaying patient photographs in the EHR provides decision support functionality for enhancing patient identification and reducing WPOE errors while being noninterruptive with minimal risk of alert fatigue. Successful implementation of such a program in an ED setting involves a modest financial investment and requires appropriate engagement of patients and staff.

## Key Points

**Question:**

Can wrong-patient order entry errors be reduced with noninterruptive display of patient photographs?

**Findings:**

In this cohort study involving 2 558 746 orders that were placed for 71 851 patients, displaying a patient’s photograph in the banner of the electronic health record was associated with a significant reduction in the rate of wrong-patient order entry errors. Unlike prior interventions, this solution required no added practitioner time burden or risk of alert fatigue.

**Meaning:**

The results of this study suggest that capturing patient photographs and displaying them in the electronic health record may be a simple and cost-effective strategy for reducing wrong-patient errors.

## Teaser

This cohort study evaluates whether noninterruptive display of patient photographs in the banner of the electronic health record is associated with a decreased rate of wrong-patient order entry errors.

## Introduction

Wrong-patient order entry (WPOE) represents an important type of error. Although studies indicate that practitioners place more than 99.9% of all orders for the correct patient,^1,2,3,4^ the large number of orders placed by practitioners each day suggests that even an error rate of less than 1 in 1000 orders would still lead to approximately 600 000 orders placed for the wrong patient annually in the US.^4^ Wrong-patient order entry occurs more frequently in the emergency department (ED), with its rate estimated to be up to 2 per 1000 orders.^2^ This increased error rate may be attributable to the often crowded and fast-paced environment of the ED, where ED practitioners frequently multitask by caring for several patients at once with frequent interruptions midtask, including requests to place an order for a different patient.^5^ Unless these errors are intercepted by the ordering practitioner or another member of the care team, they can lead to patient harm, including death.^6,7^

Strategies used to reduce WPOE have focused on improving patient identification by interrupting practitioners during electronic order entry. These strategies include a patient verification alert or an electronic form in which the practitioner must enter certain patient identifiers (eg, medical record number or first and last initials) to confirm the patient’s identity.^1,2^ Alerts can also include warnings about patients with similar names because this issue is associated with a higher risk of WPOE errors.^8^ However, these interruptive solutions risk slowing practitioners’ workflow, and multiple exposures to electronic alerts can reduce the practitioners’ engagement with the alerts, also known as alert fatigue.^9^ Therefore, noninterruptive solutions for enhancing patient identification and reducing wrong-patient errors, if effective, would be desirable and important.

One such strategy is to display the patient’s photograph in the electronic health record (EHR). This solution is based on evidence that humans are good at recognizing familiar faces.^10^ In fact, a previous study^11^ has already explored the use of patient photographs in the EHR in relation to WPOE errors. However, this study^11^ used voluntarily reported wrong-patient errors as the outcome and could not provide strong evidence about this association.

In our study, we used a validated approach for measuring WPOE errors to determine whether capturing patient photographs in the ED and displaying them in the EHR was associated with a lower rate of WPOE errors. We took advantage of the fact that many commercial EHR systems can display the patient’s photograph in the EHR banner. In the redesigned ED workflow, a patient’s photograph was recorded at the time of ED registration, and we measured the success of this initiative over time and based on various patient factors. Our goal was to evaluate the impact of passively displayed patient photographs on WPOE errors. We also sought to provide insight into factors associated with successful implementation of a patient photographs campaign.

## Methods

### Study Design and Setting

**Figure 1.**
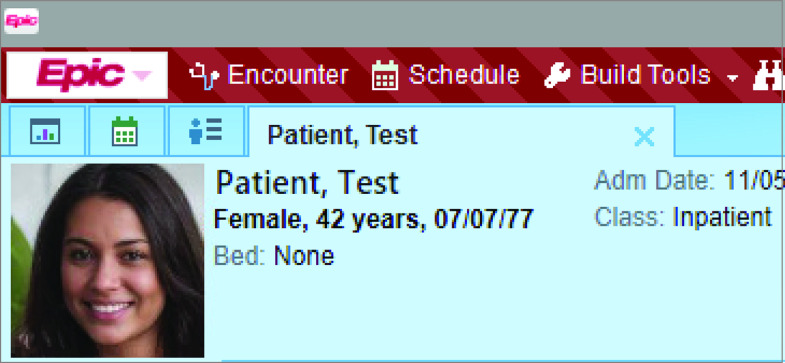
Patient Photograph Feature in the Epic Electronic Health Record Demographic data are not from a real patient. The image of the face is also not of a real human; it was generated using Style Generative Adversarial Network (owned by Nvidia). The screenshot is used with permission from Epic Systems Corporation.

We performed a quasi-experimental study with a historical cohort design in an urban, academic ED in Boston, Massachusetts, which has 59 acute care and observation beds and an annual volume of approximately 60 000 patient visits; our data set included patients who were seen in the ED between July 1, 2017, and June 31, 2019. The ED serves adult patients and supports emergency medicine residency and fellowship programs. Patient registration workflow is standard for all patients, including walk-ins and ambulance arrivals. A single instance of the Epic EHR (Epic Systems) is used across all ED, inpatient, and ambulatory areas; the patient photograph feature of Epic was available and enabled when it was installed at this institution (Figure 1). Informed consent was waived because this was a secondary, retrospective analysis of data produced as part of hospital operational initiatives. Data were deidentified after they were obtained and before the analysis. The evaluation of the association of patient photographs with WPOE errors was conducted with approval from the Partners Healthcare institutional review board. The study followed the Strengthening the Reporting of Observational Studies in Epidemiology (STROBE) reporting guideline.^17^

Before this study, a small number of interested practitioners were occasionally taking photographs of their patients with their consent and uploading the photograph into the patient’s EHR. (A few patients had uploaded their own photograph through the patient portal as well.) However, uploading of photographs was not performed systematically. Therefore, the hospital conducted a quality improvement campaign to expand the capture of patient photographs in the ED. This campaign began in July 2018 and included several components: educating the ED registration staff of the importance of patient photographs for patient identification and patient safety, educating patients using posters placed in the waiting area of the ED that explained the importance of patient photographs and inviting patient participation in this campaign, supporting the registration team by providing equipment that enhanced the quality of photographs and facilitated the photograph capture process, and assisting registration managers using analytical reports and ad hoc data queries to identify trends and areas of opportunity in this process. The quality improvement campaign did not specifically focus on education of practitioners about the risk of WPOE errors or how to avoid them.

### Outcome Measure

The primary outcome measure used for this study was the rate of WPOE errors. To measure this, we used the wrong-patient retract-and-reorder (RAR) measure, which is a validated measure endorsed by the National Quality Forum.^12^ The RAR measure estimates the rate of near-miss, wrong-patient order events by querying the EHR database. An order qualified for the RAR algorithm if it was discontinued by the ordering practitioner within 10 minutes of its being placed and then reordered identically by the same practitioner for a different patient within the next 10 minutes. This measure is estimated to have a positive predictive value of more than 76.2% in identifying WPOE events.^1^ This measure has been previously used to quantify WPOE errors and to assess the impact of various risk factors and interventions on the rate of this error.^1,2,3,8,13,14,15^

### Statistical Analysis

We used orders as the unit of analysis for this study, and our primary analysis only included orders placed by practitioners when the patient was in the ED. The primary outcome measure was a dichotomous variable (ie, each order qualified or did not quality as an RAR event). Like previous publications^1,4^ on the RAR measure, we calculated and reported the RAR rate as a rate per 100 000 orders. We defined the study groups as photograph and no photograph. An order counted toward the photograph group if the patient’s photograph was available in the EHR when the order was placed because the patient’s photograph may have been acquired earlier in the same ED encounter or was previously acquired (eg, in a previous ED encounter).

In addition, we captured several covariates about patients, practitioners, and encounters. A 2-sided test with a threshold of *P* < .10 was used to select variables. The final model was created using these variables, and for this model, *P* < .05 was used as the threshold of significance. Variables included patient’s race/ethnicity, sex, and age; practitioner type and sex; the emergency severity index (ESI, with scores ranging from 1 [immediate] to 5 [nonurgent]) assigned by the triage nurse to the patient for the ED encounter in which the order was placed; ED disposition; whether the order was placed during the day shift (defined as 7 am to 7 pm) or the night shift; and the number of workspaces opened by the practitioner at the time the order was placed. Values for the demographic variables (sex and race/ethnicity) were entered by the registration staff based on patient responses. Our EHR system is configured to allow practitioners to use up to 4 workspaces (tabs) concurrently; each workspace may be a unique patient record or complementary information about the same patient. Because multitasking and interruption are particularly common in the ED, we included the number of workspaces open at the time of order entry as a proxy for multitasking.

We conducted several secondary analyses. First, in a segmented regression analysis, we compared the slope of a linear fit for the trend in overall RAR rate before vs after the initiation of the patient photograph campaign. Second, we conducted a segmented regression analysis to compare the trends in the percentage of orders placed in the ED for patients with a photograph used before vs after the initiation of the patient photograph campaign. Third, we repeated the primary multivariable analysis using order sessions as the unit of analysis. An order session was defined as a collection of orders that were consecutively placed by the same practitioner for the same patient; if any order in the session qualified for RAR, the entire session would be considered as an RAR event in this analysis.^4^ Fourth, we modified the multivariable regression model to include a random intercept for each practitioner to assess whether practitioner-level variation played a significant role in the rate of WPOE errors. We conducted all analyses using R statistical software, version 3.5.0 (R Project for Statistical Computing).^16^

## Results

We analyzed 2 558 746 orders for the primary analysis, of which 596 346 (23.3%) were placed while the patient’s photograph was displayed to the practitioner (Table 1). These orders were placed for 71 851 unique patients (mean [SD] age, 49.2 [19.1] years; 42 677 (59.4%) female; 55 109 (76.7%) non-Hispanic).

**Table 1.** Characteristics of the Study Population

	No. (%)	
Characteristic	Photograph	No photograph
**Orders**		
Total No.	596 346	1 962 400
No. of orders placed during same session, mean (SD)	4.48 (5.37)	4.72 (5.73)
Order type		
Diagnostic	251 129 (42.1)	878 868 (44.8)
Medication	196 474 (32.9)	588 897 (30.0)
Nursing	55 310 (9.3)	188 870 (9.6)
Other	93 433 (15.7)	305 765 (15.6)
Work shift		
Day	341 805 (57.3)	1 039 515 (52.9)
Night	254 541 (42.7)	922 885 (47.0)
Workspaces		
1	237 368 (39.8)	878 868 (44.8)
2	299 335 (50.2)	939 752 (47.9)
3	24 669 (4.1)	86 025 (4.4)
4	30 189 (5.1)	104 404 (5.3)
Not recorded	4785 (0.01)	14 473 (0.7)
**Encounters**		
Total No.	32 156	90 036
ED length of stay, mean (SD), h	7.14 (8.43)	7.23 (8.49)
ESI score		
1 (Immediate)	148 (0.5)	1697 (1.9)
2 (Emergency)	11 033 (34.3)	35 513 (39.4)
3 (Less urgent)	16 787 (52.2)	41 903 (46.5)
4 (Nonurgent)	3599 (11.1)	8838 (9.8)
5 (Urgent)	339 (1.0)	714 (0.8)
NA	250 (0.8)	1371 (1.5)
ED disposition		
Discharge	21 552 (67.0)	53 550 (59.5)
Admit to inpatient care	9246 (28.8)	31 904 (35.4)
Other	1358 (4.2)	4582 (5.1)
**Patients**		
Total No.	19 091	60 849
Age, mean (SD), y	48.0 (19.4)	50.3 (20.2)
Sex		
Male	7613 (39.9)	24 882 (40.9)
Female	11 478 (60.1)	36 027 (59.2)
Race		
White	10 008 (52.4)	34 814 (57.2)
Black	3673 (19.2)	11 295 (18.6)
Hispanic or Latino	1848 (9.7)	3964 (6.5)
Other^a^	3562 (18.5)	10 776 (17.7)
Ethnicity		
Hispanic	4507 (23.6)	10 852 (17.8)
Non-Hispanic	14 110 (73.9)	47 181 (77.5)
Other^b^	474 (2.5)	2816 (4.6)
**Practitioners**		
Total No.	2457	4298
Type		
Resident or fellow	1182 (48.1)	1845 (42.9)
Attending physician	713 (29.0)	1375 (32.0)
NP or PA	359 (14.6)	641 (14.9)
Other	144 (5.9)	390 (9.1)
Sex		
Male	1213 (49.4)	2198 (51.1)
Female	1238 (50.4)	2094 (48.7)
Other or unknown	6 (0.002)	6 (0.1)

Abbreviations: ED, emergency department; ESI, Emergency Severity Index; NA, not applicable; NP, nurse practitioner; PA, physician assistant.

^a^ Asian, Pacific Islander, Native American, declined, or unknown.

^b^ Declined or unknown.

Although minor differences existed between the 2 study groups in terms of the order-, encounter-, patient-, and practitioner-level characteristics, these differences were not meaningful; the only exceptions were the difference in the ESI scores between the 2 groups (patients with the highest level of acuity, ie, ESI score of 1 or 2, were significantly more likely to be in the no photograph group) and ED disposition (patients who were eventually discharged home were more likely to be in the photograph group). The last 2 variables were associated (ie, patients with lower acuity were more likely to be discharged home). It is possible that patients with lower acuity were more likely to be asked to have their photograph taken at the time of registration or were more likely to agree. We also saw a slight disproportionality among race/ethnicity groups across the 2 study groups.

### Primary Analysis

The overall rate of RAR events was 186 per 100 000 orders for the no photograph group and 133 per 100 000 orders for the photograph group. The unadjusted odds ratio (OR) of RAR events in the photograph group vs the no photograph group was 0.72 (95% CI, 0.57-0.89). Table 2 lists the covariates that were included in the final multivariable model. After adjustment for these covariates, the association between study group and RAR rate remained consistent (OR, 0.57; 95% CI, 0.52-0.61). Patients assigned an ESI score of 1 or 2 had significantly lower odds of wrong-patient orders, but a larger number of workspaces open at the time of order entry was associated with a slightly higher rate of wrong-patient orders (the mode for number of workspaces open at the time of order entry was 2). White race was also associated with a lower rate of wrong-patient errors (OR, 0.91; 95% CI, 0.84-0.98).

**Table 2.** Results of Logistic Regression Model

Variable	Odds ratio (95% CI)
Study group	
Photograph	0.57 (0.52-0.61)
No photograph	1 [Reference]
Patient	
Race	
Hispanic or Latino	0.89 (0.75-1.05)
White	0.92 (0.85-0.99)
Other	0.92 (0.82-1.03)
Black	1 [Reference]
Ethnicity	
Non-Hispanic	1.03 (0.92-1.16)
Other	1.15 (0.94-1.40)
Hispanic	1 [Reference]
Sex	
Male	1.02 (0.96-1.09)
Female	1 [Reference]
Practitioner type	
Resident or fellow	1.05 (0.98-1.13)
NP or PA	1.08 (0.98-1.18)
Other	0.96 (0.46-2.02)
Attending physician	1 [Reference]
ESI score	
1 (Immediate)	0.33 (0.25-0.44)
2 (Emergency)	0.74 (0.69-0.78)
4 (Less urgent)	1.96 (1.72-2.24)
5 (Nonurgent)	1.25 (0.67-2.33)
3 (Urgent)	1 [Reference]
Time of order	
Shift	
Night	0.94 (0.88-1.00)
Day	1 [Reference]
No. of workspaces	
1-4	1.06 (1.03-1.11)
Intercept	1 [Reference]

Abbreviations: ESI, Emergency Severity Index; NP, nurse practitioner; PA, physician assistant.

### Secondary Analyses

**Figure 2.**
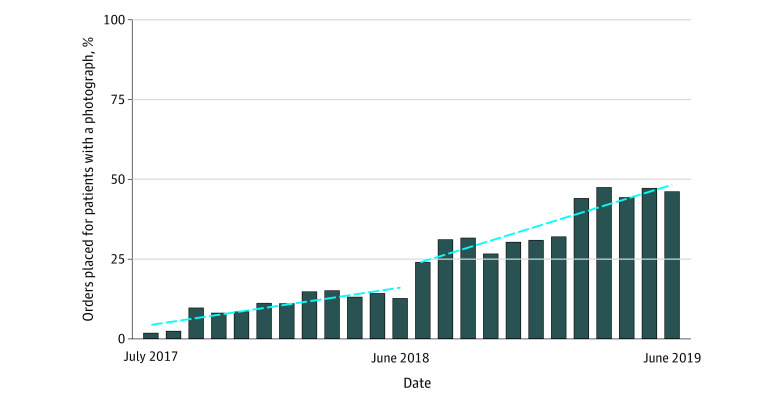
Segmented Regression Analysis of the Percentage of Orders Placed for Patients With Photographs Dashed lines represent the linear fit for each segment.

There was a slight increase in the percentage of orders placed for patients with a photograph before the start of the campaign (linear regression slope: 1.06 percentage points per month; 95% CI, 0.62-1.50 percentage points); however, this increase became significantly more pronounced after the campaign started (slope: 2.16 percentage points per month; 95% CI, 1.41-2.92 percentage points) (Figure 2). In the last 4 months of the study period, a mean (SD) of 46% (1.9) of the orders placed in the ED were entered while the patient’s photograph was displayed in the EHR.

**Figure 3.**
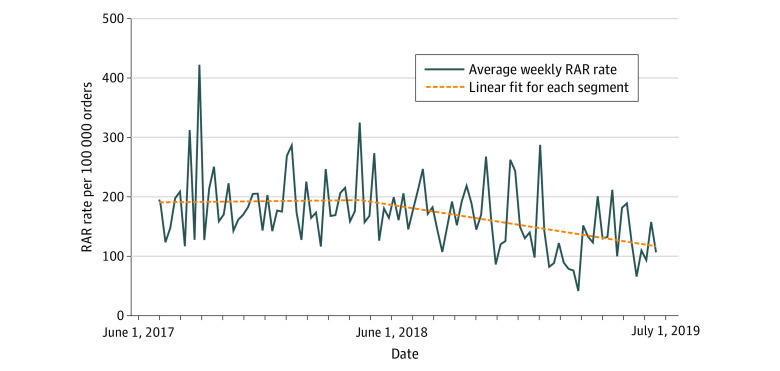
Segmented Regression Analysis of the Rate of Retract-and-Reorder (RAR) Events Before and After the Implementation of the Photographs Program Dashed lines represent the linear fit for each segment.

Despite the increasing number of patients with photographs uploaded before the initiation of the campaign, the overall rate of RAR events did not significantly improve during that period (slope: 0.006 RAR events per 100 000 orders per month; 95% CI, −0.173 to 0.185 RAR events per 100 000 orders per month) (Figure 3). In comparison, the rate of RAR events significantly decreased after the initiation of the campaign (slope: −0.189 RAR events per 100 000 orders per month; 95% CI, −0.308 to −0.070 RAR events per 100 000 orders per month).

Repeating the analyses using order sessions as the unit of analysis led to similar findings, with an unadjusted OR of 0.65 (95% CI, 0.57-0.75) and an adjusted OR of 0.65 (95% CI, 0.59-0.73). The overall rate of RAR events was 474 per 100 000 order sessions for the no photograph group and 310 per 100 000 order sessions for the photograph group.

The mixed-effects model fit the data slightly better than the fixed-effects model (Akaike information criterion of 60 166 vs 61 459), but the results remained generally consistent (OR, 0.65; 95% CI, 0.53-0.63). An ad hoc analysis using all hospital orders (N = 27 560 246) found that displaying patient photographs in the banner of EHR reduced the RAR rate in all settings, although the effect size was not as pronounced as in the ED (OR, 0.77; 95% CI, 0.75-0.80). In another ad hoc analysis, we introduced an interaction term for White race and the photograph group, but the interaction term was not statistically significant (OR, 0.91; 95% CI, 0.77-1.08).

## Discussion

In this historical cohort study, displaying patient photographs in the banner of the EHR was associated with a statistically significant reduction of WPOE errors in the ED. The effect size associated with this strategy was larger than in previously published interventions aimed at reducing WPOE errors, and this strategy has the advantage of being noninterruptive in nature.^1,2^

For detection of the reduction in WPOE errors for patients with photographs in the EHR, it is necessary that photographs be captured for many patients. Obtaining patient photographs can be difficult in the ED setting because of time pressures and because severely ill patients may not be amenable to being asked for or consenting to capturing their photographs. In these findings, patients who were acutely ill (ESI scores 1 and 2) were notably less likely to end up in the photograph group of the study, but they also have notably lower odds of wrong-patient errors. This finding could be attributable to the higher level of attention these patients receive from their practitioners, reducing the chances of a wrong-patient error. Alternatively, it is possible that the type of orders placed for critically ill patients is distinctly different from orders placed for other patients, such that practitioners are more likely to catch these errors before placing those orders for the wrong patient.

Many hospitals have moved to enterprise EHRs that share a single database instance. Photographs captured in other settings (at ambulatory clinics, at inpatient admitting offices, or by scanning the patient’s identification card at the time of creating a record) are then available and displayed in the EHR when patients are in the ED as well. Furthermore, some patient portals and mobile apps now offer the ability for patients to upload their own photographs. Emergency departments may benefit from these additional sources of patient photographs. As patients naturally age or their facial features change over time with disease, organizations may also want to have a policy of when photographs should be updated to ensure the photographs accurately reflect the patients. In addition, as health information exchange programs mature, patient photographs might be an additional data element that could be exchanged among health care organizations.

Multitasking through opening multiple workspaces at the same time is an important part of the ED practitioners’ workflow (as was evident in this study because most providers had 2 workspaces open); the association between the number of workspaces and WPOE may reflect the effect of multitasking and should not be interpreted as causal.^3^ The data in this study also suggest a weak association between White race and lower rates of WPOE, but further research is needed to explore its causality.

This study would not have been possible without high levels of engagement by the registration staff and the patients. We believe this level of engagement was attained through clear and frequent communication of the importance of patient photographs for improved patient identification and patient safety as well as feedback and continuous redesign of the involved workflows. The campaign focused on supporting easy-to-use and efficient technology for photograph capture: switching from desktop computers equipped with webcams to mobile devices (eg, Apple iPod Touch) running Epic’s Haiku mobile application. The study suggests that supporting the patient registration staff and managers with data and analytics on photograph capture rates contributed to their continuous engagement in this initiative.

Finally, this program was relatively inexpensive to implement. The costs associated with the program included the time used to train the staff on taking photographs, time spent by managers of the patient registration team to monitor photograph capture adherence and troubleshoot issues as they arose, and the cost of the equipment used for capturing photographs. In this study, we purchased 6 handheld devices and supporting accessories for a total of less than $1600. We estimate that annual operating costs for maintenance and replacement of equipment will be approximately $1000. We speculate that the expected savings from improved safety, although not measured, likely far exceed the minimal costs of this program.

### Limitations

This study has several limitations. The design of the study was quasi-experimental^18^; patients, practitioners, and orders were not randomized in terms of the display of patient photographs; practitioners could not be blinded to the study groups; and the 2 study groups had significant differences in terms of patient acuity. Despite these limitations, several factors support a causal interpretation of the association of patient photographs and lower rates of wrong-patient errors, including the larger reduction in wrong-patient errors once photographs of more patients were captured (ie, dose-response effect), the persistence of the association after adjustment for key covariates, and the consistent results of this study compared with previous research^3^ in which the association of number of workspaces and rate of wrong-patient errors was studied. The current study was a single-center study, and results may be different at other institutions, particularly those using a different EHR system with a different way of displaying patient photographs. Both of these limitations can be addressed through a multicenter, block-randomized study.

In addition, this study used the RAR measure to capture WPOE errors, but this measure depends on self-intercepted near-miss wrong-patient errors and cannot serve as a criterion standard. Although this measure has a high positive predictive value, its sensitivity is unknown. Nevertheless, this measure is superior to what was used in previous research^11^ of patient photographs (ie, voluntarily reported WPOE errors), and the use of near-miss errors in safety research is endorsed by major organizations that study or promote patient safety.^19,20,21^ Moreover, our study focused on only WPOE errors, but other types of wrong-patient errors, such as writing notes on the wrong patient or reviewing the wrong patient’s data, may also be mitigated once patient photographs are used.^22^

Finally, we did not incorporate time-dependent variables in our analyses. If patient photographs indeed impact practitioners’ likelihood of wrong-patient errors, it is possible that this effect would be subject to a learning process (ie, the effect size might increase over time); on the other hand, many forms of electronic decision support have found to be subject to alert fatigue (ie, a reduction of effect size over time), including interventions that were used to reduce wrong-patient errors.^2^

## Conclusions

In this study, displaying patient photographs in the EHR provided decision support functionality for enhancing patient identification and reducing WPOE errors while being noninterruptive with minimal risk of alert fatigue. Successful implementation of such a program in an ED setting involves a modest financial investment and requires appropriate engagement of patients and staff.
